# Molecular and Phenotypic Expansion of Alström Syndrome in Chinese Patients

**DOI:** 10.3389/fgene.2022.808919

**Published:** 2022-02-08

**Authors:** Qianwen Zhang, Yu Ding, Biyun Feng, Yijun Tang, Yao Chen, Yirou Wang, Guoying Chang, Shijian Liu, Jian Wang, Qian Li, Lijun Fu, Xiumin Wang

**Affiliations:** ^1^ Department of Endocrinology and Metabolism, Shanghai Children’s Medical Center, School of Medicine, Shanghai Jiao Tong University, Shanghai, China; ^2^ Department of Clinical Epidemiology and Biostatistics, Shanghai Children’s Medical Center, School of Medicine, Shanghai Jiao Tong University, Shanghai, China; ^3^ Department of Medical Genetics and Molecular Diagnostic Laboratory, Shanghai Children’s Medical Center, School of Medicine, Shanghai Jiao Tong University, Shanghai, China; ^4^ Center for Brain Science, Shanghai Children’s Medical Center, School of Medicine, Shanghai Jiao Tong University, Shanghai, China; ^5^ Department of Anatomy and Physiology, Ministry of Education-Shanghai Key Laboratory of Children's Environmental Health in Xinhua Hospital, Shanghai Jiao Tong University School of Medicine, Shanghai, China; ^6^ Department of Cardiology, Shanghai Children’s Medical Center, School of Medicine, Shanghai Jiao Tong University, Shanghai, China

**Keywords:** alström syndrome, ALMS1, next-generation sequencing, rare disease, variants

## Abstract

Alström syndrome (ALMS) is a rare inherited metabolic disease and ciliopathy. Large cohorts of ALMS are lacking around the world. Detailed genetic and phenotypic data were obtained from all affected individuals. Olfactory function was evaluated by the Chinese Smell Identification Test and facial pattern was analyzed with Face2gene. Fifty ALMS patients were included in this study, aged from 0.3 to 21.7 years old. Sixty-one *ALMS1* variants in 50 patients from 47 different families were confirmed, including 59 truncating and two exon deletions. Twenty-four of those variants were novel. We also summarized all previously reported cases of Chinese ALMS patients (69 patients) and identified specific and common variants within the Chinese population. Besides, the Chinese Smell Identification Test scores in patients was lower than that in controls (11.97 Vs. 10.44, *p* < .05), indicating olfactory identification impairments in ALMS patients. The facial pattern in ALMS patients was also distinctive from that of the controls (*p* < .05). In conclusion, this is the largest cohort of Chinese ALMS patients. We have successfully identified both specific and common variants in our cohort. We found a new phenotype of olfactory impairments in ALMS patients through a case-control study.

## Introduction

Alström syndrome (ALMS; MIM #203800), a ciliopathy caused by mutations of the *ALMS1* gene, is inherited in an autosomal recessive pattern. The incidence of this disease is approximately 1/1,000,000 (Tahani et al., 2020). ALMS is a complex multisystem disease whose main symptoms include retinal dystrophy, hearing loss, early-onset obesity, cardiomyopathy, type 2 diabetes mellitus, and multiple organ fibrosis. Retinal dystrophy occurs in all ALMS patients and is a critical manifestation that can be detected from a few weeks to 6 months of age. Hearing loss usually presents with progressive bilateral sensorineural hearing loss, and obesity is usually observed within 6 months to 2 years of age alongside an increased appetite. There are two forms of cardiomyopathy: infantile- and later-onset cardiomyopathy, both of which vary in severity and prognosis. Infantile-onset cardiomyopathy is severe and usually transient; however, later-onset cardiomyopathy is progressive and has a poor prognosis (Tahani et al., 2020).

The *ALMS1* gene is located in chromosome two at locus 2p13 and spans 23 exons. Most disease-causing variants of *ALMS1* are truncating mutations that result in loss-of-function proteins (Marshall et al., 2015). To date, 278 variants of *ALMS1* have been identified according to The Human Gene Mutation Database with the mutational hotspots being in exons 8, 10, and 16. There have been no disease-causing variants identified in exons 1, 2, 6, 7, 13, 22, and 23 thus far (Marshall et al., 2015). Such variants were mainly reported by studies of ALMS patient cohorts from Turkey, American, and Poland (Ozantürk et al., 2015; Zmyslowska et al., 2016; Brofferio et al., 2017). However, although there are a few case reports of ALMS patients in China (Rethanavelu et al., 2020), large-scale cohort studies of Chinese patients are still lacking, making it difficult to summarize the common features of ALMS in China.

The ALMS1 protein is located in the basal body of the primary cilia and plays an important role in ciliary function. Therefore, ALMS is a ciliopathy (Hearn et al., 2005). The defective ALMS1 protein is believed to affect primary cilia function in ALMS patients (Hearn et al., 2005; Heydet et al., 2013). Primary cilia are present in most mature mammalian cells. They are essential in many organs such as the eyes, inner ears, and hypothalamus, which may explain the phenotypes of retinal dystrophy, hearing loss, and obesity in ALMS patients. Additionally, primary cilia contribute to olfactory sensory neuron function. Olfactory dysfunction has been identified in other ciliopathies such as Bardet-Biedl syndrome (BBS) (Kulaga et al., 2004). However, it has not been reported in ALMS patients.

Here, we report the clinical and genetic spectrum of 50 ALMS patients in China, which is, to our knowledge, the largest ALMS cohort in East Asia. In total, we identified 61 variants in the *ALMS1* gene, including 50 truncating variants and two exon deletions, of which 24 are novel. Notably, we also identified the first variant c.1415_1416insTCCT in exon 7. Furthermore, genotype-phenotype correlations were analyzed, and the phenotypes of ALMS patients in China were compared to those found in three cohorts from Turkey, American, and Poland. We also summarized all of the variants identified in the Chinese ALMS patients (*n* = 69) from our study and previous reports. We identified distinct and common variants within the Chinese patients. To expand on new phenotypes and explore new diagnostic tools, we further designed a cases-control study to assess the facial patterns and the olfactory function of ALMS patients, which may be used as auxiliary diagnostic methods for ALMS.

## Materials and Methods

### Patients

A total of 50 patients, aged from 0.3 to 21.7 years old, were recruited through the Alström Syndrome Greater China Association, a patient-centered group for ALMS (https://www.alstrom.cn/) for genetic consulting in Shanghai Children’s Medical Center. They were diagnosed with ALMS at different districts throughout China. Phenotypic and genetic data were obtained from all affected individuals, including general information, medical history, personal history, inspection reports, and laboratory reports. Diagnosis was then confirmed by professional physicians at Shanghai Children’s Medical Center. Obesity was defined as BMI ≥28 kg/m^2^ and overweight was defined as BMI ≥24 kg/m^2^ for adults according to the criteria from Working Group on Obesity in China (WGOC) (Pan et al., 2021). For children between 6 and 18 years old, overweight/obesity was defined according to Chinese reference values released by the National Health and Family Planning Commission of the People’s Republic of China (China, N.H.C.o.t.P.s.R.o., 2018; Liu, 2021). Obesity in children less than 5 years was defined as a weight-for-height ≥ 2 standard deviation (SD) and overweight ≥3 SD above the WHO Growth Reference median (https://www.who.int/news-room/fact-sheets/detail/obesity-and-overweight). And in children aged 5,6 years, obesity was defined as a BMI-for-age greater than two SD above the WHO Growth Reference median; and overweight is greater than one SD above the WHO Growth Reference median (https://www.who.int/news-room/fact-sheets/detail/obesity-and-overweight). Written informed consent was obtained from all the participants or their guardians before information was collected.

### Genetic Sequencing

For patients 36 and 42–46, exome sequencing was performed at Shanghai Children’s Medical Center as previously described (Hu et al., 2018; Li et al., 2019). Candidate variants were screened by a minor allele frequency <1% against the 1,000 Genomes Project, the NHLBI exome variant server or in 50 HapMap control exomes. Then retinal degeneration, obesity, and cardiomyopathy were selected as the filtering clinical symptoms to further analyze those variants. Next-generation sequencing (NGS) in other patients were performed through other commercial companies or hospitals. Variants detected were confirmed by Sanger sequencing in each proband and their parents except for patients 35, 42, 44, and 45. The potential pathogenicity of the variant was evaluated with PolyPhen-2 (http://genetics.bwh.harvard.edu/pph2/), SIFT (http://sift.jcvi.org/), and MutationTaster (http://www.mutationtaster.org/ChrPos.html). The allele frequencies of all identified variants were much lower than 0.1%. All variants were re-identified by the geneticists at Shanghai Children’s Medical Center according to the guidelines recommended by the American College of Medical Genetics and Genomics (ACMG) (NM_015120.4).

### Automated Image Analysis

Face2Gene (https://face2gene.com) was used as a tool to analyze phenotypic traits based on pattern recognition of frontal photographs of the patients. Because Face2Gene’s CLINIC application was not yet designed to differentiate ALMS patients, we used the Face2Gene RESEARCH application to analyze facial features (Knaus et al., 2018).

### Olfactory Identification Assessment

ALMS patients and healthy children (controls) were included if the following criteria were met: 1) aged 6–18 years; 2) willing to participate in this study; 3) no difficulty in language communication. Cases and controls were excluded if they had a history of respiratory allergies, upper respiratory infections, rhinitis within the past 14 days, smoking or drinking. Controls with any chronic diseases were also excluded.

The smell identification test includes 16 different odorized items selected from the Chinese Smell Identification Test (CSIT), which has been used as a validated tool to assess olfactory function in the Chinese people (Feng et al., 2019; Wu et al., 2019). The test was conducted in a quiet room free of odors and with proper ventilation. During the CSIT, felt-tip pens containing different odorants were placed 2 cm below the individuals’ noses for 2 s after removing the cap. Participants were requested to distinguish the correct smell from four different odor options. Each correct choice was scored one point; otherwise, 0 point was recorded. Each individual only had one chance to complete the test to avoid smell fatigue. After the CSIT, a self-reported olfactory scale was obtained based on a 5-point scale.

### Statistics Analysis

Qualitative data are expressed as frequency (%) and compared using Chi-squared test of Fisher test, quantitative data showed as mean ± SD and comparisons were performed by unpaired *t*-test or nonparametric tests where appropriate. SPSS 25.0 (Statistical Package for the Social Sciences Inc., Chicago, IL, United States) was used for statistical analyses. *p* < .05 was considered statistical significance with two-sides.

## Results

### Clinical Manifestations

**FIGURE 1:**
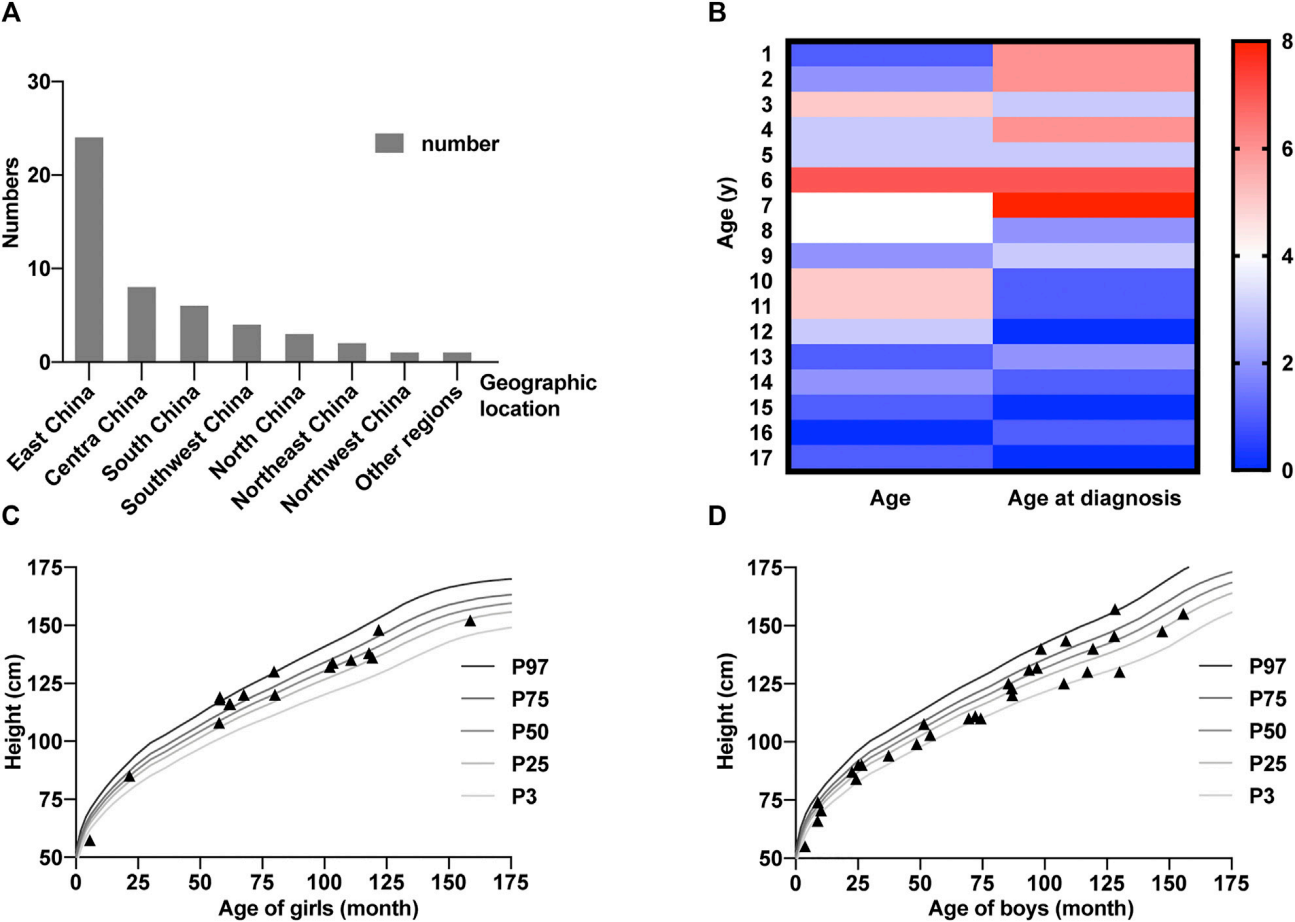
Basic characters of the Chinese cohort. **(A)** Numbers of patients in different areas of China. **(B)** Heatmap representing age distribution of 50 ALMS patients. The columns represent the number of patients with ages at present and at diagnosis. The color bar shows the range of numbers. **(C,D)** Height of girls **(C)** and boys **(D)** included in this study. The standard child growth curves are extracted from Li et al. (2009).

A total of 50 patients from 47 different families were included in this study (19 women and 31 men, Supplementary Table S1). Families 17, 21, and 47 each had two siblings. All probands from a non-consanguineous family are of Han nationality, except for patients 49 and 50, who are of Tibetan nationality. Among them, sixteen patients (patients 1–8, 10–13, 15, 19, 21–22) were previously reported by Rethanavelu et al. (Rethanavelu et al., 2020) and one patient (patient 9) was reported by Xu et al., (2016). Most patients are from East China, Central China, and South China (Figure 1A) and younger than 10 years of age (Figure 1B). Patient heights are almost close to normal ranges according to the Chinese Growth Reference (Figures 1C,D) (Li et al., 2009). Furthermore, the distribution of height among girls is higher than that among boys.

Detailed clinical manifestations of the patients are summarized in Supplementary Table S1. The average age at onset (vision impairment or cardiomyopathy) is 0.7 years, and the average age at diagnosis is 4.7 years. Visual impairment, including nystagmus, photophobia, and impaired vision, is the first symptom in 72% of the patients, and the remaining 28% of patients are referred to the hospital for infantile-onset cardiomyopathy. Overall, one-third of the patients (18/49, 37%) have a history of cardiomyopathy. Obesity and hepatic symptoms are observed in more than 50% of the patients. The prevalence of diabetes mellitus (DM), late-onset cardiomyopathy, and renal symptoms is lower than that of other symptoms (9%, 8%, and 20% respectively).

### Phenotypic Comparison Between Our Chinese Cohort and Cohorts From Other Countries

To understand whether the major clinical manifestations of ALMS are consistent across patients worldwide, we compared phenotypes between our Chinese cohort and three cohorts from other countries: Turkey, America, and Poland (Ozantürk et al., 2015; Zmyslowska et al., 2016; Brofferio et al., 2017) (Table 1). The overall clinical features were similar between these cohorts. Vision loss, the typical symptom of ALMS, occurred in all patients from all four cohorts. There were also no significant differences in the incidence of overweight status, cardiomyopathy, and hepatic symptoms between these cohorts. However, we observed significant differences in the incidence of sensorineural hearing loss (SNHL), DM, and renal symptoms between the Chinese cohort and the other cohorts. This could be because of differences in patient ages with an average of 7.0 years in the Chinese cohort and an average of 15.1 years in the other cohorts (Table 1). Thus, the different clinical symptoms could occur or become more severe as age increases. In addition, many patients in the Chinese cohort lacked the necessary renal function and liver function evaluations, which could have resulted in the incidence of associated symptoms being underestimated. In general, we believe that the major clinical symptoms are common for ALMS patients in the world.

**TABLE 1:** Features of our Chinese cohort compared with other cohorts.

Features	Chinese cohort (N = 50)	Turkish cohort^g^ (N = 33)	American’s cohort (N = 38)	Polish cohort (N = 12)
Age (mean ± SD, y)	6.95 ± 4.42	**15.83 ± 9.19** ^***^ **^a^**	**16.04 ± 10.46** ^***^ **^a^**	**13.50 ± 6.08** ^***^ **^a^**
Gender (M/F)	31/19	18/15^b^	18/20^b^	8/4^c^
Obesity^h^, n/N (%)	33/49 (67)	12/33 (36)^**^ ^b^	24/38 (63)^b^	**12/12 (100)** ^*^ **^d^**
Overweight^h^, n/N (%)	38/49 (78)	27/33 (82)^b^	33/38 (87)^b^	ND
Vision, n/N (%)	48/48 (100)	33/33 (100)^d^	37/37 (100)^d^	12/12 (100)^d^
SNHL, n/N (%)	19/45 (42)	**24/31 (77)** ^**^ **^b^**	**29/32 (91)** ^**^ **^c^**	7/10 (70)^c^
Cardiomyopathy, n/N (%)	18/49 (37)	ND	ND	4/12 (33)^c^
Infantile-onset Cardiomyopathy, n/N (%)	16/49 (33)	4/31 (13)^c^	13/38 (34)^b^	ND
Late-onset Cardiomyopathy, n/N (%)	4/49 (8)	4/31 (13)^c^	4/38 (11)^c^	ND
Diabetes mellitus, n/N (%)	4/47 (9)	**11/33 (33)** ^*^ **^c^**	**14/38 (37)** ^**^ **^c^**	**5/12 (42)** ^*^ **^c^**
Renal symptom, n/N (%)^e^	3/15 (20)	11/24 (46)^c^	**21/37 (57)** ^*^ **^c^**	2/12 (17)^d^
Hepatic symptom, n/N (%)^f^	17/28 (61)	26/33 (79)^b^	ND	5/10 (50)^d^

F, female; M, male; SNHL, sensorineural hearing loss; ND, no data. Bold indicates that there are significant differences between the two groups (*p* < .05).

a *p*-value by the Kruskal–Wallis test.

b *p*-value by the Pearson Chi-square.

c *p*-value by the Pearson Chi-square with continuity correction.

d *p*-value by the Fisher exact test, *p < .05, **p < .01, ***p < .001.

e Renal symptom includes proteinuria, fat liver, and elevated liver enzymes.

f Hepatic symptom includes hepatic steatosis, fat liver, and elevated liver enzymes.

g 33/44 patients from Turkish cohort were included for the including criteria of carrying at least one pathogenic/likely pathogenic variant of *ALMS1*.

h Obesity and overweight was defined as BMI ≥28 kg/m^2^ or BMI ≥24 kg/m^2^ for adults according to the WGOC, criteria. For children between 6 and 18 years old, overweight/obesity was defined according to Chinese reference values released by the National Health and Family Planning Commission of the People’s Republic of China. For patients aged 5,6 years, obesity was defined as a BMI-for-age greater than two SD, above the WHO, growth reference median; and overweight is greater than one SD, above the WHO, Growth Reference median. Obesity in children less than 5 years was defined as a weight-for-height ≥ 2 SD, and overweight ≥3 SD, above the WHO, growth reference median.

### Spectrum of *ALMS1* Variants in Chinese Patients

**FIGURE 2:**
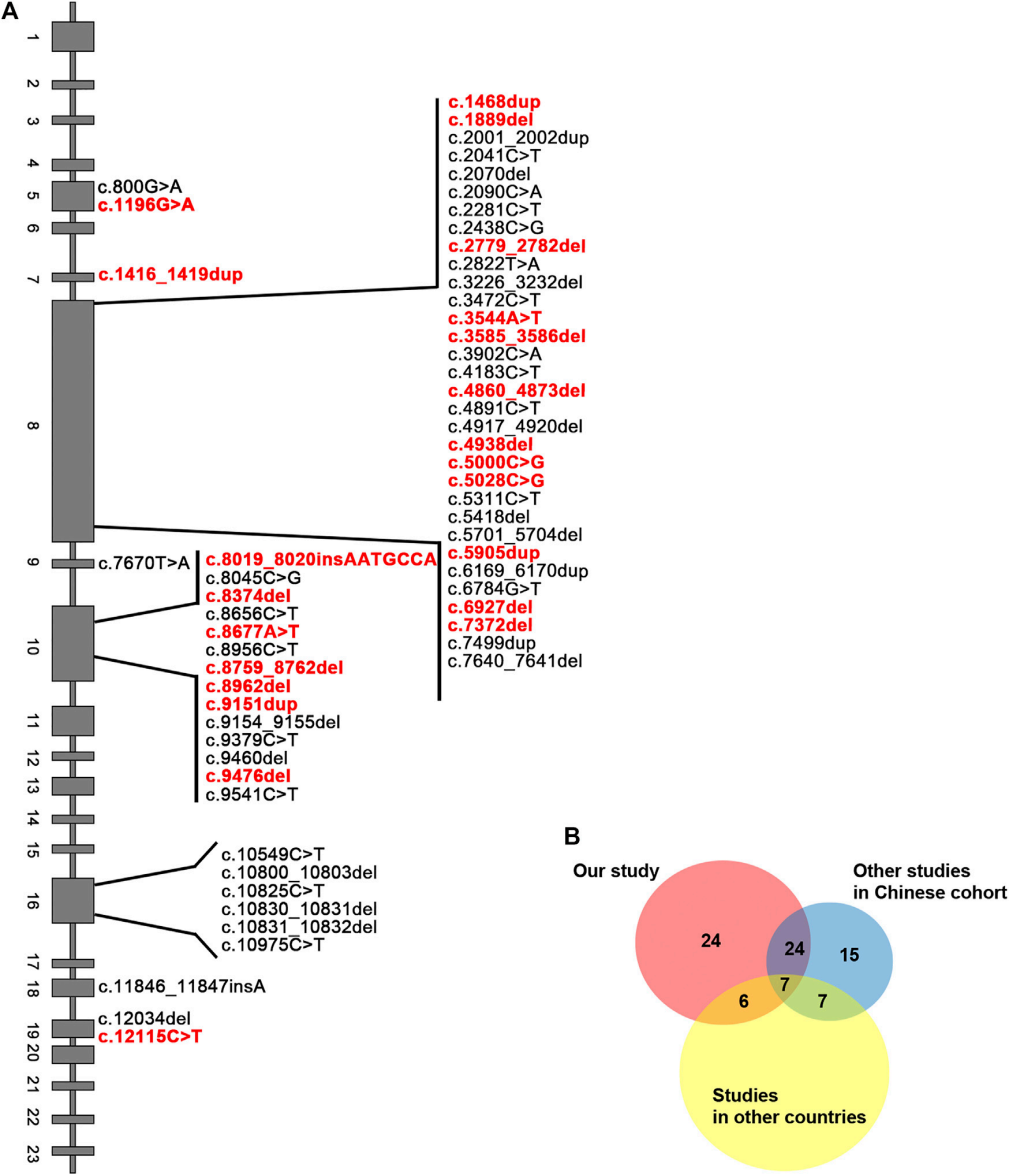
Summary of *ALMS1* variants. **(A)** *ALMS1* variants identified in this study. Novel variants are shown in red. **(B)** Pie chart for the number of variants identified in our study and other cohorts.

Next, we sought to define the variant spectrum of *ALMS1* in the Chinese ALMS patients, aiming to identify the prevalent variants in patients globally, and the distinct variants in the Chinese patients. We totally identified 61 different variants of *ALMS1* (59 truncating variants and two exon-deletions) in total in the 50 patients using exome sequencing (Figure 2A). The allele frequencies of these variants were <0.1% against the 1,000 Genomes Project and the Exome Aggregation Consortium databases. Additionally, a variety of in silico studies were conducted to evaluate the pathogenicity of these variants. According to the guidelines recommended by ACMG, all of the 59 truncating variants were classified as pathogenic. Among them, twenty-four were novel and are located in exons 5, 7, 8, 10, and 19 (Table 2 and Figure 2A). Notably, we identified the first *ALMS1* variant in exon 7, i.e., c.1415_1416insTCCT. The remaining 37 variants have been reported previously in other cohorts (Minton et al., 2006; Flintoff and Josifova, 2007; Liang et al., 2013; Consugar et al., 2015; Marshall et al., 2015; Ozantürk et al., 2015; Wang et al., 2015; Rethanavelu et al., 2020). To better understand the commonality of those 37 variants, we summarized the variants reported by other Chinese cohorts and cohorts from other countries. We retrieved 24 Chinese ALMS patients from 22 families from the literature (Liu et al., 2009; Liang et al., 2013; Wang et al., 2015; Xu et al., 2016; Yang et al., 2017; Rethanavelu et al., 2020; Zhou et al., 2020). All variants of the *ALMS1* gene from 69 Chinese families are summarized in Table 3 and the distribution of these variants in Chinese and other countries’ cohorts are shown in Figure 2B. Twenty-four variants were only identified in our cohort and 23 variants were identified in both our cohort and the other Chinese cohorts. Of them, ranking variants included c.2090C > A (11/138, 7.97%), c.10825C > T (9/138, 6.52%), c.10831_10832del (5/138, 3.62%), c.4917_4920del (5/138, 3.62%), c.3902C > A (5/138, 3.62%), and c.6169_6170dup (4/138, 2.9%). Variant c.9154_9155del (7/138, 5.07%) was recurrent in our cohort but was not found in the other cohorts. Interestingly, c.2090C > A was a distinct variant in the Chinese patients and only identified in them. Based on our analysis, exons 8, 10, and 16 were mutational hotspots, harboring 51.45%, 20.29%, and 18.84% of variants, respectively. Exon 16 had a particularly high mutation rate considering its size (Table 4). Generally, we found that the spectrum of *ALMS1* variants in Chinese ALMS patients showed clusters in some exons (8, 10, and 16) and specific variants.

**TABLE 2:** Novel variants in *ALMS1* identified in this study.

Exon	Nucleotide change	Amino acid change
5	c. 1196G > A	p. W399*
7	c. 1,416_1419dup	p. K474Sfs*2
8	c. 1468dup	p. T490Nfs*15
8	c. 1889del	p. G630Vfs*12
8	c. 2779_2782del	p. L927Sfs*6
8	c. 3544A > T	p. K1182*
8	c. 3585_3586del	p. K1198Tfs*12
8	c. 4860_4873del	p. L1621Hfs*15
8	c. 4938del	p. A1647Lfs*44
8	c. 5000C > G	p. S1667*
8	c. 5028C > G	p. Y1676*
8	c. 5905dup	p. S1969Ffs*19
8	c. 6927del	p. T2310Rfs*34
8	c. 7372del	p. T2458Qfs*20
10	c. 8019_8020insAATGCCA	p. D2674Efs*4
10	c. 8374del	p. G2792Vfs*22
10	c. 8677A > T	p. K2893*
10	c. 8759_8762del	p. P2920Lfs*19
10	c. 8962del	p. L2988Ffs*8
10	c. 9151dup	p. T3051Nfs12
10	c. 9476del	p. I3159Nfs*3
19	c. 12115C > T	p. Q4039*
	Exon 8 del
	Exon 17-21del	

**TABLE 3:** Summary of *ALMS1* variants in 69 Chinese ALMS families (47families in this study and 22 families in previous reports).

Exon	Nucleotide change	Amino acid change	No. of alleles	Allele frequency (%)	References
**Novel variants identified in this study**					
5	c. 800G > A	p. W267*	1	0.72	This study
5	c. 1196G > A	p. W399*	1	0.72	This study
7	c. 1415_1416insTCCT	p. A472Afs*4	1	0.72	This study
8	c. 1467_1468insA	p. T490Nfs*15	1	0.72	This study
8	c. 1889delG	p. G630Vfs*12	1	0.72	This study
8	c. 2779_2782delCTTT	p. L927Sfs*6	1	0.72	This study
8	c. 2822T > A	p. L941*	2	1.45	This study
8	c. 3544A > T	p. K1182*	1	0.72	This study
8	c. 3585_3586delAA	p. K1198Tfs*12	1	0.72	This study
8	c. 4183C > T	p. Q1395*	1	0.72	This study
8	c.4860_4873delACTTGGAGAGAAGC	p. L1621Hfs*15	1	0.72	This study
8	c. 4938delA	p. A1647Lfs*44	1	0.72	This study
8	c. 5000C > G	p. S1667*	1	0.72	This study
8	c. 5028C > G	p. Y1676*	1	0.72	This study
8	c. 5905_5906insT	p. S1969Ffs*19	1	0.72	This study
8	c. 6927delC	p. T2310Rfs*34	1	0.72	This study
8	c. 7372delA	p. T2458Qfs*20	1	0.72	This study
10	c. 8019_8020insAATGCCA	p. D2674Efs*4	1	0.72	This study
10	c. 8045C > G	p. S2682*	1	0.72	This study
10	c. 8374delG	p. G2792Vfs*22	1	0.72	This study
10	c. 8656C > T	p. R2886*	1	0.72	This study
10	c. 8677A > T	p. K2893*	1	0.72	This study
10	c. 8759_8762delCTTC	p. P2920Lfs*19	1	0.72	This study
10	c. 8959delC	p. P2987Pfs*9	1	0.72	This study
10	c. 9152dupA	p. T3051Nfs*12	1	0.72	This study
10	c. 9379C > T	p. Q3127*	1	0.72	This study
10	c. 9460delG	p. V3154*	2	1.45	This study
10	c. 9476delT	p. I3159Nfs*3	1	0.72	This study
19	c. 12115C > T	p. Q4039*	2	1.45	This study
	Exon 8 del		1	0.72	This study
	Exon 17-21del		2	1.45	This study
**Known variants identified in previous reports but not in this study**					
5	c. 805C > T	p. R269*	1	0.72	Wang et al. (2015)
5	c. 1054C > T	p. R352*	1	0.72	Xu et al. (2016)
8	c. 2994_2995delAG	p. T996Tfs*9	1	0.72	Zhou et al. (2020)
8	c. 3181C > T	p. Q1061*	1	0.72	Wang et al. (2015)
8	c. 3727A > T	p. K1243*	1	0.72	Rethanavelu et al. (2020)
8	c. 5049dupA	p. P1684Tfs*2	1	0.72	Rethanavelu et al. (2020)
8	c. 5631dupA	p. G1878Rfs*7	1	0.72	Xu et al. (2016)
8	c. 6305C > A	p. S2102*	1	0.72	Xu et al. (2016)
8	c. 6436C > T	p. R2146*	1	0.72	Yang et al. (2017)
8	c. 6823C > T	p. R2275*	1	0.72	Rethanavelu et al. (2020)
8	c. 7402G > T	p. E2468*	1	0.72	Xu et al. (2016)
8	c. 7436C > G	p. S2479*	2	1.45	Liang et al. (2013)
10	c. 8041G > T	p. E2681*	1	0.72	Rethanavelu et al. (2020)
10	c. 8335C > T	p. Q2471*	2	1.45	Liu et al. (2009)
10	c. 8782C > T	p. R2928*	1	0.72	Xu et al. (2016)
10	c. 9441_9442insAATA	p. Q3147Qfs*2	1	0.72	Liang et al. (2013)
10	c. 9448insA	p. N3150Kfs*2	1	0.72	Liang et al. (2013)
10	c. 9535C > T	p. R3179*	1	0.72	Zhou et al. (2020)
16	c. 10883insG	p. R3611Efs7*	1	0.72	Liang et al. (2013)
16	c. 11015delA	p. N3672Ifs11*	1	0.72	Liang et al. (2013)
16	c. 11107C > T	p. R3703*	1	0.72	Liang et al. (2013)
16	c. 11110_11128del	p. R3704Lfs*11	3	2.17	Wang et al. (2015)
**Common variants in this study and previous reports**					
8	c. 1995_1996insCT	p. T666Lfs*7	1	0.72	This study and Rethanavelu et al. (2020)
8	c. 2041C > T	p. R681*	3	2.17	This study and Xu et al. (2016)
8	c. 2070delT	p. D691Ifs*4	2	1.45	This study and (Wang et al., 2015)
8	c. 2090C > A	p. S697*	11	7.97	This study and Liang et al. (2013), Wang et al. (2015), Xu et al. (2016)
8	c. 2281C > T	p. Q761*	1	0.72	This study and Rethanavelu et al. (2020)
8	c. 2438C > G	p. S813*	1	0.72	This study and Rethanavelu et al. (2020)
8	c. 3226_3232delAAAGTTT	p. K1076Qfs*10	1	0.72	This study and Rethanavelu et al. (2020)
8	c. 3466C > T	p. Q1156*	1	0.72	This study and Rethanavelu et al. (2020)
8	c. 3902C > A	p. S1301*	5	3.62	This study and (Yang et al., 2017)
8	c. 4891C > T	p. Q1631*	2	1.45	This study and Xu et al. (2016)
8	c. 4917_4920delTAAA	p. N1639Kfs*4	5	3.62	This study and Xu et al. (2016), Rethanavelu et al. (2020)
8	c. 5311C > T	p. Q1771*	1	0.72	This study and Rethanavelu et al. (2020)
8	c. 5418delC	p. Y1807Tfs*23	2	1.45	This study and Rethanavelu et al. (2020)
8	c. 5701_5704_delGAGA	p. E1901Rfs*18	2	1.45	This study and Xu et al. (2016)
8	c. 6169_6170dupAT	p. L2058Ffs*17	4	2.90	This study and Xu et al. (2016)
8	c. 6784G > T	p. E2262*	1	0.72	This study and Rethanavelu et al. (2020)
8	c. 7499dupT	p. L2501Tfs*25	1	0.72	This study and Rethanavelu et al. (2020)
8	c. 7640_7641delAG	p. E2547Vfs*9	1	0.72	This study and Rethanavelu et al. (2020)
9	c. 7670T > A	p. L2557*	1	0.72	This study and Rethanavelu et al. (2020)
10	c. 8653C > T	p. Q2885*	1	0.72	This study and Rethanavelu et al. (2020)
10	c. 9154_9155delCT	p. C3053Sfs*9	7	5.07	This study and Rethanavelu et al. (2020)
10	c. 9541C > T	p. R3181*	1	0.72	This study and Rethanavelu et al. (2020)
16	c. 10549C > T	p. Q3517*	3	2.17	This study and Rethanavelu et al. (2020)
16	c. 10800_10803delTGAA	p. E3601Cfs*60	1	0.72	This study and Rethanavelu et al. (2020)
16	c. 10825C > T	p. R3609*	9	6.52	This study and Rethanavelu et al. (2020)
16	c. 10830_10831delGA	p. R3611Afs*6	1	0.72	This study and Rethanavelu et al. (2020)
16	c. 10831_10832delAG	p. R3611Afs*6	5	3.62	This study and Xu et al. (2016)
16	c. 10975C > T	p. R3659*	1	0.72	This study and Rethanavelu et al. (2020)
18	c. 11846_11847insA	p. N3952Lfs*10	1	0.72	This study Rethanavelu et al. (2020)
19	c. 12034delC	p. L4012Wfs*19	1	0.72	This study and Rethanavelu et al. (2020)

**TABLE 4:** Allele rate and distribution of *ALMS1* variants in different exons from 69 Chinese ALMS families.

Exon	Exon size (bp)	Number of alleles	Allele rate^a^ (%)
5	473	4	0.8
7	94	1	1.1
8	6108	71	1.2
9	134	1	0.7
10	1865	28	1.5
16	1,163	26	2.2
18	204	1	0.5
19	242	3	1.2

a Allele rate = Number of alleles/Exon size.

### Genotype-Phenotype Correlations

Interestingly, the siblings from family 17 suffered from severe infantile-onset cardiomyopathy, which indicated the possibility of a genotype-phenotype correlation. Therefore, we tried to find genotype-phenotype correlations in the ALMS patients. We analyzed patients with a history of infantile-onset cardiomyopathy and compared them to patients without a history of cardiomyopathy. In total, 32% (16/50) of the patients suffered from infantile-onset cardiomyopathy in our cohort. Of these patients, 93.75% (15/16) carried at least one truncated variant before exon 8 (including exon 8); only 67.65% (23/34) of the patients without cardiomyopathy had similar truncated variants (Table 5). However, no significant statistical difference was identified (*p* = .097), likely because of the limited number of patients. These results suggest that earlier translation termination may be correlated with infantile-onset cardiomyopathy and that the fragment encoded by exon 8 may be important for cardiac function.

**TABLE 5:** Genotype-Phenotype correlation for Infantile-onset cardiomyopathy ALMS patient.

Subjects	Patients with a history of infantile-onset cardiomyopathyN = 16	Patients without a history of infantile-onset cardiomyopathyN = 34	*p* Value
Age (mean ± SD, y)	5.84 ± 4.01	7.46 ± 4.39	.201^a^
Gender (male/female)	8/8	23/11	.230^b^
Carrying variants before exon 8, n (%)	15 (93.75)	23 (67.65)	.097^c^

a *p*-value by the Mann-Whitney *U* test.

b *p*-value by the Pearson Chi-square.

c *p*-value by the Pearson Chi-square with continuity correction. *p < .05.

### Computer Facial Recognition Analysis

**FIGURE 3:**
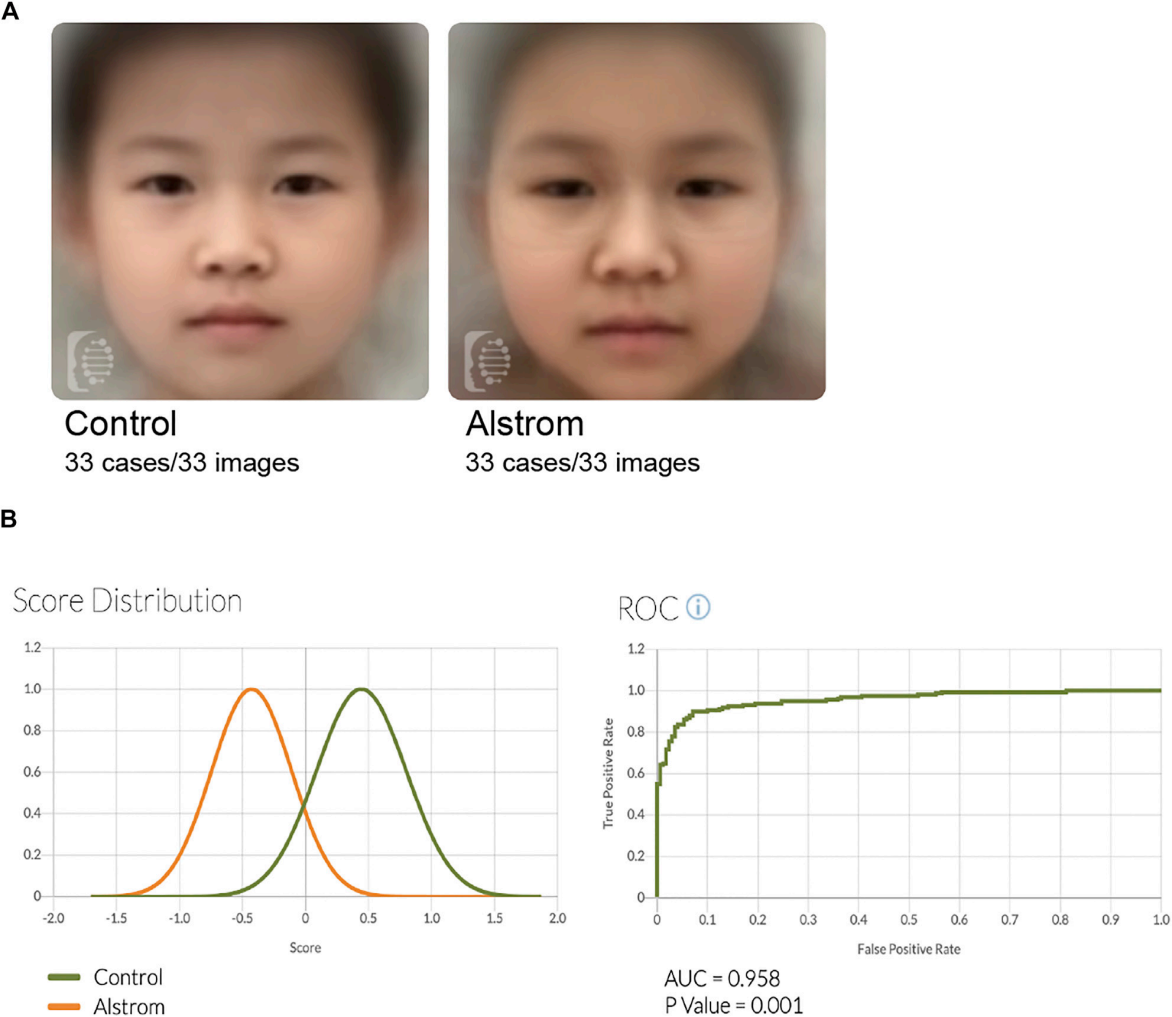
Facial analysis of patients and controls. **(A)** Composite photos computed from age-matched images of ALMS patients and controls. **(B)** Score distribution and ROC curves of the comparison results of ALMS patients and controls.

Next, we wondered if we could develop novel methods which might aid in the diagnosis of ALMS. Firstly, we tested whether ALMS patients had distinct facial features. We used the Face2Gene RESEARCH tools to assess patient facial patterns. Thirty-three facial photographs of ALMS patients were collected, along with those from 33 age- and sex-matched controls. The receiver operating characteristic (ROC) curves showed that the facial patterns in ALMS patients were indeed different from those in the controls, with an Area Under Curve (AUC) of 0.96 (*p* < .001) (Figures 3A,B). This suggests that the facial recognition analysis could be an auxiliary diagnostic method for ALMS.

### Olfactory Assessment

**FIGURE 4:**
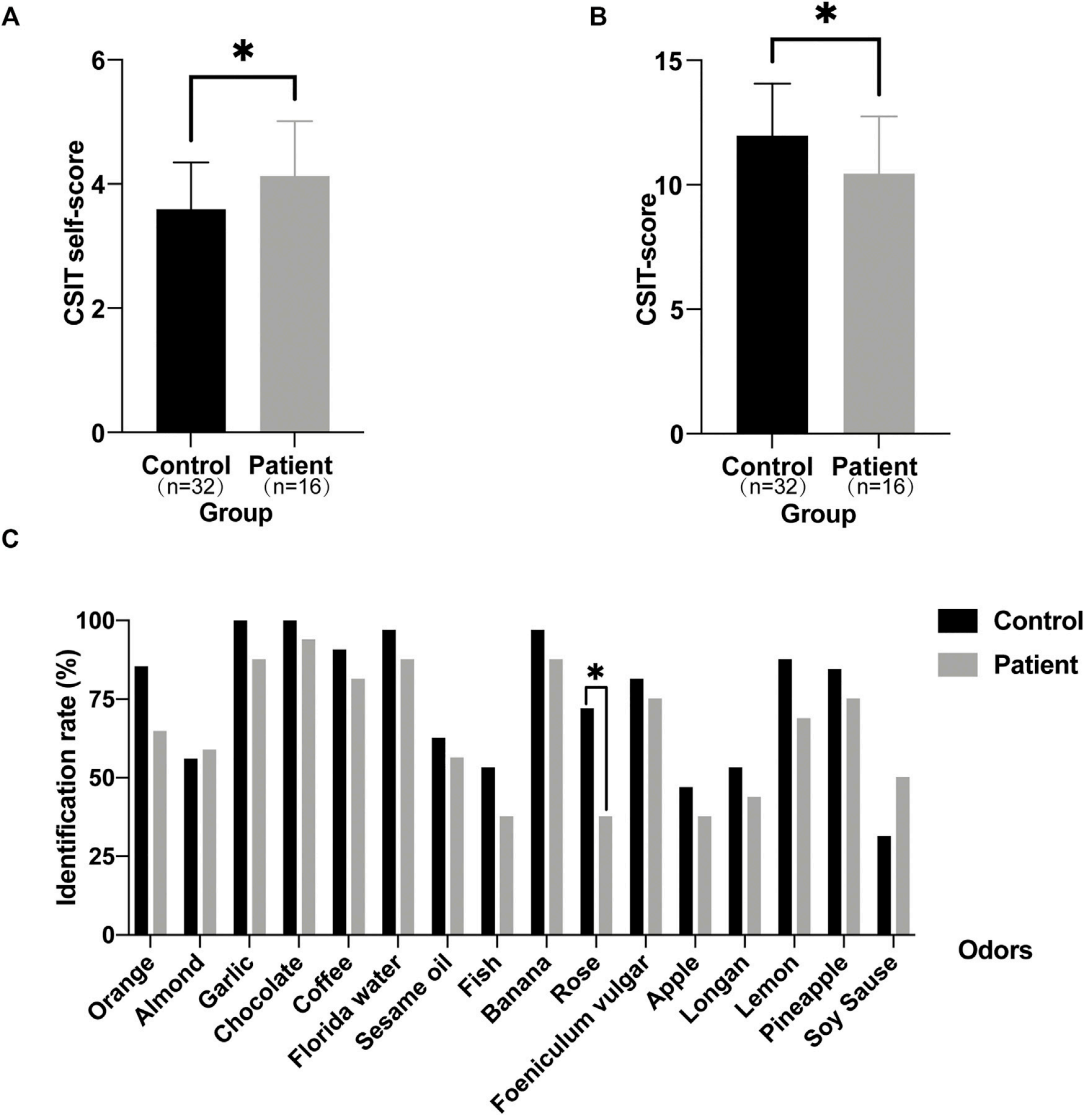
Olfactory assessment in ALMS patients and controls. **(A)** The CSIT self-scores of ALMS patients and controls. **(B)** The tested CSIT scores of ALMS patients and controls. **(C)** The identification rate of each odor in ALMS patients and controls. *p < .05.

Next, we tended to find other potential diagnosis method. *ALMS1* variants have been reported to result in defective cilia structures and trafficking of key signaling molecules in the cilia (Hearn et al., 2005; Heydet et al., 2013). Therefore, ALMS patients have visual and auditory dysfunctions. Interestingly, olfactory sensory neurons detect volatile odorants using olfactory receptors and other signaling molecules located in the cilia. However, it is unknown if olfactory function is normal in ALMS patients. We used CSIT, a toolkit specifically designed to assess olfactory function in the Chinese population, to evaluate olfactory function in ALMS patients (Feng et al., 2019; Wu et al., 2019). Sixteen patients were recruited and were matched with 32 individual controls by age and sex. The average age of patients was 9.50 ± 2.28 years and of controls, 9.63 ± 2.12 years. Although the CSIT self-score was higher in patients than that in controls, the tested CSIT score in patients was 10.44 in patients and 11.97 in controls. The slightly lower but significant CSIT score in ALMS patients indicated that olfactory identification (OI) impairments occurred in ALMS patients (Figures 4A,B). Further analysis showed that, for most odors, the identification rate was slightly lower in patients than that in controls. Only the identification rate of rose odor in the patients was significantly lower in patients than that in the controls (Table 6 and Figure 4C). We further compared the differences in age, sex, early-onset cardiomyopathy, and genotypes between ALMS patients who could identify the rose odor and those who could not (Table 7). Unfortunately, no differences between the two groups were found. It should be noted that the patients and controls included in this study were very young and may not have been able to describe the odors correctly, which could have resulted in the olfactory impairment in ALMS patients being underestimated. In conclusion, the olfactory assessment could be used as another auxiliary diagnosis method for ALMS.

**TABLE 6:** Detailed information of Chinese Smell Identification Test in ALMS patients and controls.

Smell	Patients N = 16 (%)	Control N = 32 (%)	*p* Value
Orange	10 (64.71)	27 (85.29)	.089^c^
Almond	10 (58.82)	18 (55.88)	.679^c^
Garlic	14 (87.50)	32 (100.00)	.106^b^
Chocolate	15 (93.75)	32 (100.00)	0.333^b^
Coffee	13 (81.25)	29 (90.63)	.643^d^
Florida water	14 (87.50)	31 (96.88)	.527^d^
Sesame oil	9 (56.25)	20 (62.50)	.676^c^
Fish	6 (37.50)	17 (53.13)	.307^c^
Banana	14 (87.50)	31 (96.88)	.527^d^
Rose	6 (37.50)	23 (71.88)	**.022** ^*^ **^c^**
Foeniculum vulgar	12 (75.00)	26 (81.25)	.900^d^
Apple	6 (37.50)	15 (46.88)	.537^c^
Longan	7 (43.75)	17 (53.13)	.540^c^
Lemon	11 (68.75)	28 (87.50)	.239^d^
Pineapple	12 (75.00)	27 (84.38)	.695^d^
Soy Sauce	8 (50.00)	10 (31.25)	.206^c^

Data are shown as the number of patients and controls that successfully identified the odors. The identification rates are calculated as the percentages and are placed inside the parentheses. Bold indicates that there are significant differences between the two groups (*p* < .05).

b *p* value by the Fisher exact test.

c *p* value by the Pearson Chi-square.

d *p* value by the Pearson Chi-square with continuity correction. *p < .05.

**TABLE 7:** Comparison analysis between ALMS patients who could identify the rose odor and those could not.

Subject	Group 1^c^N = 10	Group 2^d^N = 6	*p* Value
Gender (M/F)	7/3	3/3	.089^a^
Age (mean ± SD, y)	9.74 ± 1.95	9.54 ± 3.47	.792^b^
Obesity^h^, n/N (%)	7/10 (70)	5/6 (83)	1.000^a^
Overweight^h^, n/N (%)	9/10 (90)	6/6 (100)	1.000^a^
Vision, n/N (%)	10/10 (100)	6 (100)	1.000^a^
SNHL, n/N (%)	8/10 (80)	3/6 (50)	.299^a^
Infantile-onset Cardiomyopathy, n/N (%)	3/10 (30)	1/6 (17)	1.000^a^
Late-onset Cardiomyopathy, n/N (%)	2/10 (20)	0/6 (0)	.500^a^
Cardiomyopathy, n/N (%)	5/10 (50)	1/6 (17)	.307^a^
Diabetes mellitus, n/N (%)	1/10 (10)	0/6 (0)	1.000^a^

a *p* value by the Fisher exact test.

b *p* value by the Mann-Whitney *U* test.

c Group 1 refers to patients who could identify the rose odor and.

d Group 2 refers to patients who could not identify the rose odor.

## Discussion

In this study, we recruited 50 Chinese ALMS patients for genetic and phenotypic analyses. To the best of our knowledge, this is the largest cohort study of Chinese ALMS patients. The average age of this Chinese cohort is also the youngest in the world of all ALMS cohorts. Most of the patients in this study were diagnosed before 7 years old, possibly owing to the rapid development of NGS in China. Furthermore, the time from age at onset to age at diagnosis was approximately 4 years in our cohort compared to approximately 13 years in the Polish cohort (Zmyslowska et al., 2016).

Our study has revealed that exons 8, 10, and 16 are mutational hotspots, which is consistent with previous reports (Marshall et al., 2015). However, previous studies directly sequenced exons 8, 10, and 16, whereas we performed NGS without exon bias. The preference of *ALMS1* variants in those exons is partly due to the relatively larger sizes of these exons. Among these three exons, exon 16 has the highest mutation rate (Table 4). Further, ethnicity greatly contributed to the distribution of the *ALMS1* variants. According to our study, c.2090C > A (11/138, 7.97%) in exon eight is the most common variants in China. The high carrier rate of this variant in East Asians (0.006%) may account for this pattern (Marshall et al., 2015). Other recurrent variants included: c.2041C > T (3/138, 2.17%), c.3902C > A (5/138, 3.62%), c.4917_4920del (5/138, 3.62%), and c.6169_6170dup (4/138, 2.90%) in exon 8; c. 9154_9155del (7/138, 5.07%) in exon 10, and c. 10549C > T (3/138, 2.17%), c. 10825C > T (9/138, 6.52%), and c. 10831_10832del (5/138, 3.62%) in exon 16. Of those 8 recurrent variants, c. 2041C > T, c. 2090C > A, c. 3902C > A, c. 6169_6170dup, c. 9154_9155del, and c. 10831_10832del are specific to Chinese patients, whereas c.10825C > T is common worldwide. Regarding recurrent variants in other countries, c.8177_8187del, c.8164C > T, and c.10945G > T are specific to patients of West Asian/Middle Eastern kindreds, whereas c.10775del (24/557) and c.11449C > T (15/557) are specific to Northern European and American populations (Marshall et al., 2015).

ALMS symptoms either develop or become more severe with age; thus, diagnosis is difficult during the early ages of patients. Consequently, exploring new diagnostic tools is necessary. In this study, we explored the possibility of two auxiliary diagnostic methods. The first is computer facial recognition, which has been successfully applied in diagnosis of some diseases, such as Noonan syndrome with characteristic facial features (Li et al., 2019). Facial patterns in ALMS patients are rarely mentioned, likely because of the low incidence of this disease. Since we had 50 patients, we performed facial analysis. We showed that there are significant differences in facial patterns between ALMS patients and age- and sex-matched controls. However, further studies are still required to identify the distinctive facial features of ALMS patients, which will greatly benefit the clinical diagnosis of ALMS, especially in juveniles. The second is olfactory assessment. We used the CSIT that was specifically developed for Chinese populations to evaluate the olfactory function in patients. We observed olfactory identification impairments in ALMS patients. However, we could not exclude the possibility that the patients were simply unfamiliar with the odors due to their limited exposures to them. In addition, we found that the ALMS patients particularly struggled to recognize the rose odor instead of other odors. This may suggest that ALMS1 is expressed in subsets of olfactory sensory neurons that detect distinct volatile odors. We will continue to follow-up with these patients and perform computer facial recognition and olfactory assessment annually, which may further validate our findings as the patients grow up.

In summary, we reported 50 Chinese ALMS patients with 61 pathogenic variants, including 24 novel variants. We identified recurrent variants of 138 variants in 69 Chinese ALMS families totally. Olfactory identification impairments can occur in ALMS patients, and these patients have some distinctive facial features. Early-onset cardiomyopathy is likely related to earlier transcription termination of the *ALMS1* gene. Our study expands on the *ALMS1* gene genetic and phenotypic spectrum and provides new insights into the understanding of ALMS.

## Data Availability

The data that support the findings of this study are available from the corresponding authors upon reasonable request.
