# Supplementary material for: Molecular and Phenotypic Expansion of Alström Syndrome in Chinese Patients
Source: Front Genet. 2022 Feb 8;13:808919. doi: 10.3389/fgene.2022.808919 (PMC8861322; doi:10.3389/fgene.2022.808919)
Supplement: Supplementary file 1 [file DataSheet1.PDF]

**Table S1**. Detailed characters of 50 ALMS patients

| Patient | Family | Gender | Age（y） | Age at onset | Age at diagnosis | First symptom | Retinal dystrophy | SNHL | Infantile-onset Cardiomyopathy | Late-onset Cardiomyopathy | DM | Obesity [a] | Overweight [a] | Renal symptom [b] | Hepatic symptom [c] |
|---|---|---|---|---|---|---|---|---|---|---|---|---|---|---|---|
| 1 | 1 | F | 9.8 | 1.5 | 6.2 | VI | Y | Y | N | N | N | Y | Y | ND | Y |
| 2 | 2 | M | 6 | 1 | 2.5 | VI | Y | Y | N | N | N | Y | Y | N | N |
| 3 | 3 | F | 8.6 | 0.1 | 5.4 | CM | Y | Y | Y | N | N | Y | Y | N | N |
| 4 | 4 | M | 10.8 | 0.4 | 6 | CM | Y | Y | Y | Y | Y | N | N | Y | Y |
| 5 | 5 | M | 7.2 | 0.3 | 2.6 | VI | Y | N | Y | N | N | Y | Y | ND | ND |
| 6 | 6 | M | 13 | 0.8 | 10.3 | VI | Y | Y | N | N | N | N | Y | ND | Y |
| 7 | 7 | M | 9.8 | 1.2 | 2.9 | VI | Y | N | N | N | N | Y | Y | N | Y |
| 8 | 8 | F | 5.6 | 0.3 | 1.3 | CM | Y | Y | Y | N | N | Y | Y | N | Y |
| 9 | 9 | M | 10.7 | 0.6 | 9.1 | VI | Y | N | N | N | N | Y | Y | ND | ND |
| 10 | 10 | M | 7.2 | 1.6 | 3.4 | VI | Y | N | N | N | N | Y | Y | ND | ND |
| 11 | 11 | M | 10.7 | 1.4 | 6.4 | VI | Y | Y | N | Y | N | N | Y | Y | Y |
| 12 | 12 | M | 9 | 1.4 | 5 | VI | Y | Y | N | N | N | N | N | N | Y |
| 13 | 13 | M | 8.1 | 0.8 | 4.5 | VI | Y | Y | N | N | N | Y | Y | ND | Y |
| 14 | 14 | F | 9.2 | 2 | 5.6 | VI | Y | Y | N | N | N | N | N | ND | ND |
| 15 | 15 | F | 10.2 | 0.4 | 7.7 | VI | Y | Y | N | N | N | Y | Y | ND | Y |
| 16 | 16 | M | 6.2 | ND | 3.6 | VI | Y | Y | N | N | N | Y | Y | ND | ND |
| 17 | 17 | M | 10 | 0.1 | 7.6 | CM | Y | Y | Y | N | N | Y | Y | N | Y |
| 18 | 17 | F | ND | 0.1 | 0.4 | CM | Y | ND | Y | N | ND | ND | ND | ND | ND |
| 19 | 18 | F | 5.2 | 0.5 | 3.3 | CM | Y | N | Y | N | N | Y | Y | ND | ND |
| 20 | 19 | F | 9.9 | 0.2 | 8.4 | CM | Y | N | Y | N | N | Y | Y | N | ND |
| 21 | 20 | F | 4.8 | 0.2 | 3.2 | CM | Y | N | Y | N | N | Y | Y | N | N |
| 22 | 21 | M | 7.8 | 0.7 | 6.4 | VI | Y | N | N | N | N | N | Y | ND | ND |
| 23 | 22 | M | 4.1 | 0.6 | 2.7 | VI | Y | N | N | N | N | N | N | ND | ND |
| 24 | 23 | M | 3.1 | 1.2 | 1.7 | VI | Y | N | N | N | N | Y | Y | ND | ND |
| 25 | 24 | M | 5.8 | 0.3 | 4.7 | VI | Y | N | N | N | N | N | N | ND | ND |
| 26 | 25 | F | 8.5 | 0.1 | 7.1 | VI | Y | Y | N | N | N | Y | Y | ND | Y |
| 27 | 26 | M | 7.1 | 2 | 6.4 | VI | Y | N | N | N | N | Y | Y | ND | ND |
| 28 | 27 | M | 0.8 | 0.3 | 0.2 | CM | Y | N | Y | N | N | N | N | ND | ND |
| 29 | 21 | M | 21.7 | 0 | 20.3 | VI | Y | Y | N | N | Y | N | Y | ND | Y |
| 30 | 28 | M | 2 | 0.5 | 0.3 | VI | Y | N | N | N | N | Y | Y | N | N |
| 31 | 29 | M | 0.7 | 0.1 | 0.1 | CM | Y | N | Y | N | N | N | N | N | N |
| 32 | 30 | M | 9 | 0.3 | 5.8 | VI | Y | Y | N | N | N | Y | Y | ND | ND |
| 33 | 31 | F | 6.6 | 0.5 | 5.3 | VI | Y | N | N | N | N | Y | Y | ND | Y |
| 34 | 32 | M | 2.2 | 0.6 | 0.7 | CM | Y | N | Y | N | N | N | N | ND | N |
| 35 | 33 | F | 1.8 | 0.4 | 1.2 | VI | Y | N | N | N | N | N | Y | ND | ND |
| 36 | 34 | M | 4.3 | 0.8 | 3.9 | VI | Y | Y | N | N | N | Y | Y | N | Y |
| 37 | 35 | F | 13.2 | 1.2 | 12.1 | VI | Y | Y | Y | Y | N | Y | Y | ND | Y |
| 38 | 36 | M | 1.9 | 0.3 | 0.8 | VI | Y | N | N | N | N | Y | Y | ND | ND |
| 39 | 37 | M | 2.1 | 0.7 | 1.3 | VI | Y | N | N | N | N | Y | Y | ND | ND |
| 40 | 38 | F | 0.5 | 0.1 | 0.1 | CM | Y | N | Y | N | N | N | N | N | N |
| 41 | 39 | M | 4.5 | 0.8 | 4.3 | VI | Y | N | N | N | N | Y | Y | ND | ND |
| 42 | 40 | M | 12.3 | 0.8 | 11.7 | VI | Y | N | N | N | Y | Y | Y | ND | N |
| 43 | 41 | M | 0.3 | 0.1 | 0.3 | CM | Y | ND | Y | N | N | Y | Y | ND | N |
| 44 | 42 | F | 18.1 | 2 | 12.8 | VI | Y | Y | N | Y | Y | Y | Y | Y | Y |
| 45 | 43 | M | 0.7 | 0.2 | 0.7 | CM | Y | Y | Y | N | N | Y | Y | ND | N |
| 46 | 44 | F | 5.1 | 1 | 2.1 | VI | Y | ND | N | N | N | Y | Y | ND | N |
| 47 | 45 | F | 6.7 | 0.2 | 6.1 | VI | Y | N | N | N | ND | Y | Y | ND | ND |
| 48 | 46 | M | 8.2 | 0.2 | 6.7 | VI | Y | ND | N | N | ND | Y | Y | ND | Y |
| 49 | 47 | F | 4.8 | 1 | 4.7 | VI | Y | N | N | N | N | N | N | ND | ND |
| 50 | 47 | F | 4.8 | 1.8 | 4.7 | VI | Y | N | N | N | N | N | N | ND | ND |

F, female; M, male; SNHL, sensorineural hearing loss; DM: diabetes mellitus; ND, no data; Y: yes; N: no; VI: Visual impairment, including nystagmus, photophobia, and impaired vision; CM: Cardiomyopathy.

[a] Obesity was defined as a BMI ⩾ 28 $kg/m^2$ and overweight was defined a BMI ⩾ 24 $kg/m^2$ Chinese WGOC for adults. For children between 5 to 19 years old, obesity was defined as a BMI-for-age greater than 2 standard deviations above the WHO Growth Reference median; and overweight is greater than 1 standard deviations above the WHO Growth Reference median. Obesity in children less than 5 was defined as a weight-for-height ≥ 2 SD and overweight ≥ 3 SD

[b] Renal symptom includes proteinuria and abnornal renal function.

[c] Hepatic symptom includes hepatic steatosis, fat liver, and elevated liver enzymes.
